# Gender-Based Differences in Biomechanical Walking Patterns of Athletes Using Inertial Sensors

**DOI:** 10.3390/jfmk10010082

**Published:** 2025-02-27

**Authors:** Elina Gianzina, Christos K. Yiannakopoulos, Georgios Kalinterakis, Spilios Delis, Efstathios Chronopoulos

**Affiliations:** 1 School of Physical Education and Sport Science, National and Kapodistrian University of Athens, 17232 Athens, Greece; 2 Second Department of Orthopaedics, Medical School, National and Kapodistrian University of Athens, 11527 Athens, Greece

**Keywords:** inertial sensor, biomechanical data, gait analysis, gender difference

## Abstract

**Background**: Wearable inertial sensors are essential tools in biomechanics and sports science for assessing gait in real-world conditions. This study explored gender-based differences in biomechanical walking patterns among healthy Greek athletes using the BTS G-Walk system, focusing on key gait parameters to inform gender-specific training and rehabilitation strategies. **Methods**: Ninety-five healthy athletes (55 men, 40 women), aged 18 to 30 years, participated in this study. Each athlete performed a standardized 14 m walk while 17 biomechanical gait parameters were recorded using the BTS G-Walk inertial sensor. Statistical analyses were conducted using SPSS to assess gender differences and left–right foot symmetry. **Results**: No significant asymmetry was found between the left and right feet for most gait parameters. Men exhibited longer stride lengths (left: *p* = 0.005, Cohen’s d = 0.61; right: *p* = 0.009, Cohen’s d = 0.53) and longer stride and gait cycle durations (left: *p* = 0.025, Cohen’s d = 0.52; right: *p* = 0.025, Cohen’s d = 0.53). Women showed a higher cadence (*p* = 0.022, Cohen’s d = −0.52) and greater propulsion index (left: *p* = 0.001, Cohen’s d = −0.71; right: *p* = 0.001, Cohen’s d = −0.73), as well as a higher percentage of first double support (*p* = 0.030, Cohen’s d = −0.44). **Conclusions**: These findings highlight the impact of biological and biomechanical differences on walking patterns, emphasizing the need for gender-specific training and rehabilitation. The BTS G-Walk system proved reliable for gait analysis, with potential for optimizing performance, injury prevention, and rehabilitation in athletes. Future research should explore larger, more diverse populations with multi-sensor setups.

## 1. Introduction

Gait serves as a significant indicator of overall health [1,2]. It can be used to predict various health-related outcomes, including cognitive decline [3], fall risk [4], life quality [5], and lifespan [6]. Consequently, gait analysis has gained prominence as a reliable method for assessing multiple health dimensions in both clinical and research contexts [7,8].

Traditionally, gait research has focused on populations with conditions like osteoarthritis and cerebral palsy, or on healthy older adults. These studies often examine lower limb biomechanics, including joint kinetics, spatiotemporal parameters (e.g., stride length, cadence), and joint kinematics (e.g., joint angles, range of motion) [9,10]. However, these analyses typically rely on laboratory-based systems equipped with force plates and optical motion capture technology [11]. While such systems are highly accurate and reliable, their cost, complexity, and time-intensive nature restrict accessibility and practicality for many settings [12]. Furthermore, laboratory protocols may fail to capture natural, real-world gait patterns [13].

In contrast, wearable inertial sensors provide a portable, cost-effective alternative, enabling gait analysis in diverse and real-world environments [14,15]. These devices, known as inertial measurement units (IMUs), employ accelerometers, gyroscopes, and magnetometers to measure linear accelerations and angular velocities. By attaching IMUs to specific body parts, researchers can capture biomechanical data with reliability comparable to traditional optical systems [16]. The adoption of wearable sensors for gait analysis has expanded rapidly in recent years [11,17], with studies highlighting their potential to enhance treatment planning, patient management, and healthcare efficiency [18].

Systematic reviews comparing IMU-based gait analysis with optical motion capture systems show that IMUs provide good-to-moderate agreement for kinematic measures but can be less consistent for certain spatiotemporal parameters [19]. While some studies report moderate-to-poor agreement for these measures, others confirm that IMUs offer excellent validity and reliability for key gait parameters, such as step length, stride length, and stance time, particularly in healthy adults [16]. This suggests that IMUs perform well for certain gait metrics but may vary in accuracy depending on the specific parameter, study population, and methodology used.

Overall, reviews consistently highlight that IMUs are a reliable tool for measuring essential gait parameters and can serve as a practical alternative to lab-based motion capture, especially for real-world and clinical applications [20]. However, variability remains in parameters like gait velocity and cadence, emphasizing the need for further methodological improvements. Despite these challenges, IMUs continue to gain traction for real-time gait assessments in dynamic environments where traditional motion capture is impractical.

Despite the increasing use of IMUs, research on gender-specific gait characteristics in athletic populations remains limited. Prior studies suggest that biological and biomechanical differences between male and female athletes influence stride length, cadence, propulsion, and overall gait mechanics [21,22]. Understanding these variations is crucial for optimizing performance, reducing injury risks, and tailoring rehabilitation programs.

Thus, this study aims to investigate gender-based differences in biomechanical walking patterns among healthy Greek athletes using wearable inertial sensors. Specifically, we analyze 17 key gait parameters, including stride length, gait cycle duration, cadence, propulsion index, stance phase, swing phase, double support time, and speed, among others. These parameters were chosen because they are critical indicators of walking efficiency, stability, and athletic performance. Prior research suggests that male and female athletes exhibit distinct biomechanical patterns, influencing performance optimization and injury prevention [21,22,23]. We hypothesize that men will demonstrate longer stride lengths and gait cycles, while women will exhibit higher cadence and propulsion indices. By examining these differences, our findings aim to inform gender-specific training and rehabilitation strategies, enhancing athletic performance and reducing injury risks.

## 2. Materials and Methods

### 2.1. Participants

A total of 95 healthy athletes (55 men and 40 women), aged 18 to 30 years, participated in this study. To ensure a homogeneous athletic population, participants were required to meet specific inclusion criteria: a minimum of one year of systematic training and competition experience in their respective sports; no history of musculoskeletal, orthopedic, or neurological disorders affecting gait; no lower limb injuries or surgeries in the past six months; no diagnosed balance disorders or vestibular impairments; and the ability to walk independently without assistive devices.

Exclusion criteria included the presence of any acute or chronic medical condition affecting gait or physical performance, the use of medication that could interfere with motor function or balance, a history of injuries requiring rehabilitation within the last six months, and pregnancy in female participants, as it may influence gait mechanics.

All participants provided written informed consent after receiving a detailed explanation of the study’s objectives and procedures. This study was conducted in accordance with the ethical principles outlined in the Declaration of Helsinki and was approved by the Research Ethics Biology Committee of the School of Physical Education and Sport Science at the National and Kapodistrian University of Athens (approval number: 1306/22-09-2021).

### 2.2. Instrumentations

To evaluate the biomechanical walking pattern, a validated and reliable wireless inertial sensor was used (G-Walk, BTS Bioengineering S.p.A., Milan, Italy) [24,25,26]. The device comprises a three-axis accelerometer, magnetometer, gyroscope, and positioning system receiver. The triaxial accelerometer offers various sensitivity options with a dynamic range of ±2, ±4, ±8, and ±16 g and bandwidth ranging from 4 to 1000 Hz, utilizing 16-bit precision per axis. The triaxial magnetometers have 13-bit resolution and a dynamic range of ±1200 μT, with a bandwidth of up to 100 Hz. Additionally, the triaxial gyroscope provides 16-bit precision per axis and multiple sensitivity options of ±250, ±500, ±1000, and ±2000°/s. Its bandwidth spans from 4 to 8000 Hz.

The module also features a GPS receiver with remarkable position accuracy, offering 2.5 m accuracy up to 5 Hz or 3 m accuracy up to 10 Hz and a bandwidth of up to 10 Hz.

As for dimensions, the module measures 70 mm in length, 40 mm in width, and 18 mm in height (2.75 in × 1.57 in × 0.7 in). It can achieve an acquisition frequency of up to 1000 Hz.

To facilitate data acquisition, the module can be connected to a laptop via Bluetooth 3.0 (class 1.5) with a range of up to 60 m in line of sight.

### 2.3. Acquisition Protocol

During the test, the sensor was placed inside a semi-elastic black belt worn above the participants’ S1–S2 vertebrae. This study was conducted in three phases: (1) data collection, (2) analysis, and (3) interpretation. These phases were completed in sequence, with no need for detailed time tracking.

In the data collection phase, participants performed the 14 m walk protocol. Before starting, they were given clear instructions to ensure consistency and reliability in the test results. The test began with each participant briefly standing still to allow for stabilization. When signaled, they walked along a straight path at a steady, moderate pace. The goal was for participants to complete at least five full gait cycles (over the 14 m distance) before turning around.

Before each turn, participants paused for at least 1 s to regain stability, then turned around and paused again for 1 s before continuing in the new direction. This procedure was designed to reduce variability and maintain consistency across trials while also allowing for natural walking patterns.

Each participant completed at least one trial of the 14 m walk, with a second trial conducted if needed to ensure reliable data. In total, each participant performed at least two trials, although additional trials were permitted to confirm the consistency of their gait patterns.

### 2.4. Data Analysis

Gait data were captured via Bluetooth 3.0 and transmitted in real time to a computer. G-Studio software (Version 1.3.0) was used to process the gait data and automatically apply filters to remove noise and any discontinuities in the walking patterns. The software then calculated the spatiotemporal parameters using the algorithms built into the system.

**Figure 1:**
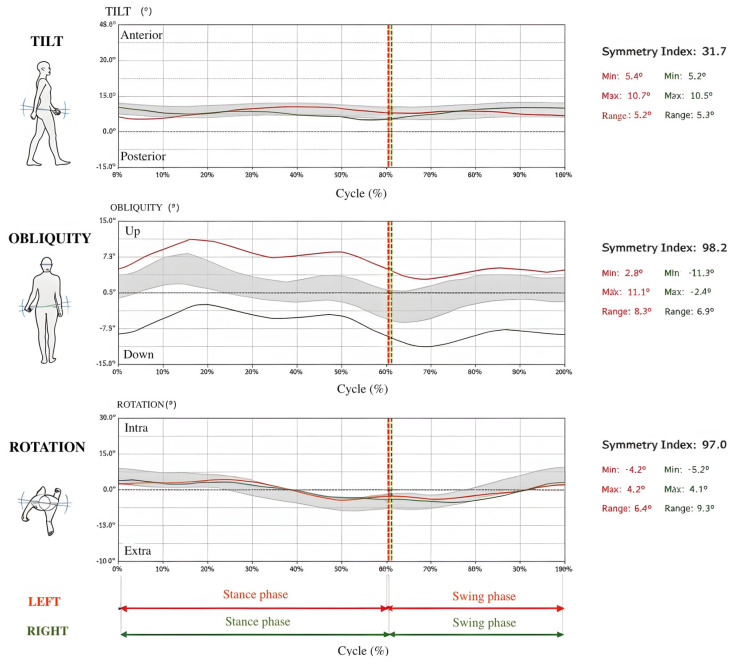
Example of patient symmetry index of pelvic angles (tilt, obliquity, rotation). Min; minimum angle. Max; maximum angle. Range; range of motion. The left and right limb movements are represented by red and green lines, respectively. The red and green dashed lines indicate the transition between stance and swing phases for the left and right limbs, respectively. The grey-shaded area represents the variability (standard deviation) of the gait parameters across the gait cycle. Symmetry indices are provided for each parameter, with minimum, maximum, and range values highlighted.

The software calculations derived several spatiotemporal parameters from the data. These parameters can be classified into two main categories, as follows:

a. Global analysis parameters:
  - “Cadence” (steps/min): Represents the number of steps taken in one minute.
  - “Speed” (m/s): Indicates the average walking speed.
  - “Symmetry Index of gait cycle”: Quantifies the percentage (%) of symmetry between the anterior/posterior acceleration curves during the right and left gait cycles.
  - “Symmetry index of pelvic angles (tilt, obliquity, rotation)”: Evaluates the percentage (%) of similarity or dissimilarity between the pelvic angles recorded during the right and left gait cycles. The pelvic angles are usually measured in three main body planes: sagittal (tilt), frontal (obliquity), and transverse (rotation) (see example in Figure 1).

b. Parameters categorized for the LEFT and RIGHT sides:
  - “Stride length” (m): Represents the average distance between each initial contact and the subsequent contact of the same side during walking.
  - “%Stride length” (%height): Represents the normalized stride length over the individual’s height.
  - “Gait cycle duration (s)”: Represents the average time interval between two consecutive heel strikes of the same foot.
  - “Step length (% str. length)”: Shows the average distance between each initial contact and the next contact made by the opposing side.
  - “Stance phase (% cycle)”: Represents the average duration of the right and left foot support phases as a percentage of the gait cycle.
  - “Swing phase (% cycle)”: Represents the average duration of the right and left swing phases as a percentage of the gait cycle.
  - “Double support phase (% cycle)”: Represents the average duration of the phase in which both feet are in stance position as a percentage of the gait cycle.
  - “Single support phase (% cycle)”: Represents the average duration of the phase in which only one foot is in stance position as a percentage of the gait cycle.
  - “Elaborated steps”: Refers to the number of strides considered in the analysis.
  - “Propulsion index”: Represents the line’s actual inclination following the acceleration pattern’s rising edge.
  - “Walk quality index”: A composite measure that quantifies the overall quality of an individual’s walking pattern by incorporating key gait parameters, such as step length, cadence, symmetry, and variability. This index provides a score that reflects the efficiency, stability, and symmetry of a person’s gait. Higher scores indicate more efficient, stable, and symmetrical walking patterns, whereas lower scores reflect less efficient, unstable, or more variable gait characteristics.

### 2.5. Statistical Analysis

IBM SPSS Statistics version 28.0 (IBM Corporation, Somers, NY, USA) was used for all statistical analyses. Descriptive statistics, including means and standard deviations (SD), were calculated for all variables. The Kolmogorov–Smirnov and Shapiro–Wilk tests were used to assess the normal distribution of the parameters. Homogeneity of variances was assessed using Levene’s test as part of the statistical analysis. The demographic characteristics of the sample were also summarized.

To evaluate gait asymmetry, a paired samples *t*-test was conducted to compare the mean values of gait parameters between the left and right foot. Additionally, to examine gender differences in gait parameters, an independent samples t-test was performed. Given the unequal sample sizes (55 men vs. 40 women), both equal variances-assumed and not-assumed conditions were considered in the analysis, with adjustments made where necessary to account for unequal variances between the two groups.

Due to the large number of gait parameters analyzed, the potential for inflated Type I error was considered. However, no formal correction (e.g., Bonferroni) was applied, as such methods can be overly conservative, potentially increasing the risk of Type II errors (false negatives) and diminishing statistical power. Instead, effect sizes (Cohen’s d) were reported alongside *p*-values to provide a more comprehensive understanding of the practical significance of observed differences.

Findings with marginal significance (0.05 > *p* > 0.01) should be interpreted with caution, as these results may reflect trends rather than conclusive evidence. The significance level for all tests was set at *p* < 0.05.

## 3. Results

During the data analysis, 17 different walking patterns were gathered from 95 individuals. Table 1 displays the demographic statistics, including mean values (SD) and *p*-values from independent samples *t*-test results, highlighting the differences between men and women.

Additionally, Table 2 displays the mean values (SD), *p*-values, and effect sizes (Cohen’s d) for the comparisons between men and women across various gait parameters. Significant differences were observed in several parameters, including cadence (*p* = 0.022, Cohen’s d = −0.52), where men exhibited a lower cadence compared to women, and left stride length (*p* = 0.005, Cohen’s d = 0.61), where men had a longer stride length. Additionally, significant differences were noted in first left double support (*p* = 0.030, Cohen’s d = −0.44), where women exhibited higher percentages of double support. Notably, the propulsion index showed a large effect size, with men demonstrating lower propulsion index scores than women (*p* = 0.001, Cohen’s d = −0.71 for the left and *p* = 0.001, Cohen’s d = −0.73 for the right).

However, several other parameters, such as speed, the symmetry index of gait cycle, and the walk quality index, did not show significant differences (*p*-values > 0.05), with small or negligible effect sizes indicating that the observed differences were likely of limited practical significance.

Finally, Table 3 displays mean values, standard deviations, *p*-values from paired *t*-test results, and effect sizes for various gait parameters. Most comparisons between left and right sides did not show significant differences, and the effect sizes were typically small (d < 0.2). However, elaborated steps (Pair 2) showed a statistically significant difference with a small effect size (d = 0.24), indicating a slight but notable difference between the left and right sides in terms of steps taken during gait. Other parameters, such as gait cycle duration, stride length, and stance duration, showed no meaningful differences, with effect sizes close to zero, reinforcing the negligible practical relevance of the differences.

## 4. Discussion

This study contributes to biomechanical gait analysis by evaluating 17 distinct walking pattern features in healthy Greek athletes using the BTS G-Walk inertial sensor system. The findings provide valuable insights into human movement mechanics, which are essential for optimizing athletic performance, injury prevention, and rehabilitation [27,28,29]. Additionally, this study demonstrates that IMUs, such as the BTS G-Walk, can accurately measure key spatiotemporal gait parameters, including step and stride lengths, swing time, and stance time, aligning with findings from systematic reviews that have confirmed the reliability of inertial sensors in both laboratory and field settings [16]. The excellent test–retest reliability observed in linear gait metrics further supports the system’s precision and makes it a promising tool for real-world applications [26].

Although IMUs showed high reliability in measuring spatiotemporal gait parameters, certain measures, particularly symmetry indices, exhibited small or negligible effect sizes, indicating minimal practical differences between the left and right sides. This finding contrasts with the literature, which has raised concerns about the variability of symmetry indices when using IMUs, particularly in clinical or performance contexts [16,19]. However, in our study, the symmetry measures were relatively stable, and the observed variability in these indices was not significant. This suggests that while concerns about symmetry metrics have been noted, our findings did not strongly confirm them. Future research should explore the conditions under which IMU-based symmetry assessments may be more or less reliable, particularly in different populations or settings.

Significant gender-based differences were observed in several gait parameters, in line with previous studies [21,22,23,30,31]. Specifically, men exhibited longer stride lengths, greater gait cycle durations, and longer stride durations, likely attributed to their greater limb length and muscle mass. These findings suggest that male athletes may have an advantage in endurance-based activities that require longer strides and more efficient energy expenditure per stride. In contrast, women demonstrated higher cadence and propulsion indices, reflecting distinct neuromuscular control strategies. This finding is particularly relevant for sports that demand quick transitions and explosive movements, such as sprinting and court-based sports [32,33,34].

However, since training status and sport type were not directly assessed in this study, these conclusions should be interpreted with caution. Further research is needed to investigate how these factors influence gait characteristics, as training regimens and sport-specific demands may have a significant effect on gait efficiency and performance. Despite these gender-based differences, no significant differences were found in speed, symmetry index, or walk quality index. This suggests that while some gender differences are evident in specific gait metrics, other factors, such as individual training status or sport specialization, may have a more substantial impact on overall gait quality and symmetry.

From an injury risk perspective, the observed gait differences align with studies suggesting gender-specific vulnerabilities. Women’s higher cadence and shorter stride lengths may lead to increased loading on the lower extremities, potentially heightening the risk of overuse injuries such as patellofemoral pain or ACL injuries [35,36]. Conversely, men’s longer stride lengths may reduce impact frequency but increase strain on the hips and lower back, possibly predisposing them to conditions such as hip impingements or lower back pain [37,38]. These biomechanical tendencies underscore the importance of personalized injury prevention strategies that account for both natural gait patterns and specific sport demands of each gender [39,40].

While this study provides a comprehensive assessment of gait characteristics in athletes, it did not directly evaluate sport-specific adaptations. Existing studies in the literature suggest that athletes in asymmetrical sports (e.g., tennis, soccer, fencing) may develop unique biomechanical traits due to their sport-specific movement patterns [41,42]. Future research should explore how these training loads, movement patterns, and sport demands influence gait asymmetries and overall biomechanics. Understanding these sport-specific adaptations could lead to more tailored training programs aimed at either correcting or capitalizing on these biomechanical features to improve performance and reduce injury risk. For example, athletes in asymmetrical sports might benefit from addressing specific gait asymmetries to enhance balance and movement efficiency [42,43].

**Figure 2:**
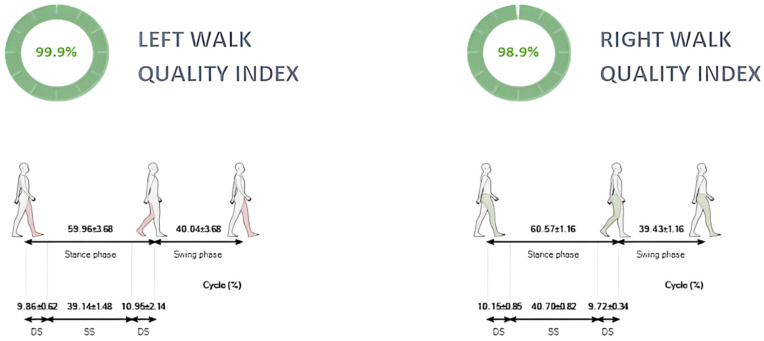
Walk analysis report. DS; double support. SS; single support.

The BTS G-Walk system demonstrated both practicality and reliability for real-world gait analysis. Its portability and user-friendly design allow for detailed assessments outside traditional laboratory settings, making it particularly useful in dynamic athletic environments (see example of walk analysis report in Figure 2). The ability to track gait metrics over time provides real-time feedback for personalized training interventions. For instance, male athletes could focus on improving stride efficiency to enhance endurance, while female athletes may benefit from refining propulsion mechanics and cadence for agility-based sports. Rehabilitation programs can use IMU-based gait tracking to monitor recovery progress, particularly for female athletes recovering from ACL injuries. Furthermore, the system’s ability to assess gait in varying terrains and footwear conditions ensures that training and rehabilitation programs can be adapted to the specific needs of athletes, based on their sport, environment, and recovery status [20].

## 5. Limitations

While the findings of this study provide valuable insights into gait mechanics, several limitations should be acknowledged.

First, the unequal sample sizes between men (*n* = 55) and women (*n* = 40) present a potential concern. This imbalance in group sizes may reduce the statistical power of the analyses, increasing the risk of both Type I and Type II errors. Although no statistical adjustments were made to account for this disparity, a post hoc power analysis indicated that a minimum of 55 participants per group would be optimal for detecting medium effect sizes with adequate power. While our current sample size is close to this estimate, future research should aim for larger and more balanced groups to improve the reliability of gender-based comparisons. Additionally, while *t*-tests were used to compare gait parameters between men and women, the assumption of equal variances was violated in some cases. Although Welch’s t-test was applied as a correction, such violations could still affect effect size estimates and overall conclusions.

Second, this study did not apply formal corrections for multiple comparisons. While this decision preserved statistical power, it also increased the risk of Type I errors due to the large number of comparisons conducted. Methods such as Bonferroni or False Discovery Rate (FDR) adjustments could help mitigate this concern in future studies and ensure more robust statistical interpretations.

Another limitation concerns the marginal significance of certain findings (0.05 > *p* > 0.01). These results may reflect trends rather than definitive evidence of a true effect, warranting cautious interpretation. Given the lack of formal corrections for multiple comparisons, the interpretation of marginal *p*-values should be approached carefully. Future studies with larger, more balanced samples and adjusted statistical thresholds would be beneficial in confirming the robustness of these trends.

This study’s cross-sectional design further restricts the ability to infer longitudinal changes or causal relationships. While this research provides a snapshot of gait parameters within a specific athletic population, longitudinal studies are needed to assess how gait mechanics evolve over time or in response to training adaptations, injuries, or rehabilitation programs.

Additionally, this study focused exclusively on healthy Greek athletes, which may limit the generalizability of the findings. The sample’s homogeneity in terms of ethnicity, culture, and athletic background may not be representative of broader populations. Future research should aim to validate these results across diverse groups, considering variations in cultural, environmental, and physiological factors to enhance the generalizability of the findings.

Furthermore, this study relied on a single-sensor system, the BTS G-Walk, which is validated for gait analysis [24,25,26]. While this system is widely used in biomechanics research and provides reliable data on key gait parameters, the findings would benefit from incorporating additional sensors (e.g., foot, knee) to offer a more comprehensive analysis of gait kinematics. A key limitation of the BTS G-Walk is its placement on the lower back, which may not fully capture joint-specific kinematics of the lower limbs. Additionally, the accuracy of IMU-derived gait parameters remains a concern, as sensor drift, misalignment, and soft tissue artifacts may introduce measurement errors. Future research using multi-sensor setups could provide a more detailed understanding of joint-specific contributions and asymmetries, helping to assess the robustness and consistency of gait measurements across different methodologies. Standardized calibration procedures and validated algorithms should also be prioritized to improve IMU-based gait assessments and reduce discrepancies between wearable and laboratory-based systems.

Addressing these limitations in future research will provide a deeper understanding of biomechanical adaptations in various athletic contexts and enhance the practical applications of gait analysis for performance optimization, injury prevention, and rehabilitation.

## 6. Conclusions

This study found significant gender-based differences in gait mechanics among healthy Greek athletes. Men exhibited longer stride lengths and gait cycle durations, while women showed higher cadence and propulsion indices. These results suggest that biological and biomechanical differences influence walking patterns, underscoring the importance of gender-specific training and rehabilitation strategies.

The BTS G-Walk system proved effective for gait analysis, with no significant left–right foot differences detected in most gait parameters, confirming its reliability. However, limitations such as sample imbalance, single-sensor use, and lack of multiple comparison corrections highlight the need for future research with larger, more diverse populations and multi-sensor setups.

Despite these limitations, the findings emphasize the potential of wearable sensors for real-world gait analysis, providing a practical tool for performance optimization, injury prevention, and rehabilitation in athletic populations.

## Figures and Tables

**Table 1:** Demographic statistics of participants (*n* = 95), with comparisons between men and women.

All Individuals (*n*: 95)			
Parameter	Men (*n*: 55)	Women (*n*: 40)	*p*-Value
Age (year)	25.13 (8.553)	23.32 (6.654)	0.349
Weight (kg)	76.58 (10.572)	61.33 (8.716)	<0.001
Height (cm)	175.62 (19.914)	166.88 (5.849)	0.010
Shoe size (EU)	43.87 (1.504)	39.38 (1.996)	<0.001

**Table 2:** Comparison of gait parameters between men and women.

Parameter	Men	Women	*p*-Value	Effect Size (Cohen’s d)
Cadence (steps/min)	107.71 (6.225)	110.65 (5.076)	0.022	−0.52
Speed (m/s)	1.30 (0.167)	1.26 (0.124)	0.178	0.27
Symmetry index of gait cycle	96.48 (1.950)	97.08 (1.567)	0.115	−0.34
Elaborated steps left	7.35 (1.126)	7.70 (0.111)	0.102	−0.44
Elaborated steps right	7.24 (1.138)	7.23 (0.961)	0.946	0.01
Stride duration left (s)	1.12 (0.064)	1.09 (0.050)	0.025	0.52
Stride duration right (s)	1.12 (0.065)	1.09 (0.048)	0.025	0.53
Gait cycle duration left (s)	1.12 (0.064)	1.09 (0.050)	0.025	0.52
Gait cycle duration right (s)	1.12 (0.065)	1.09 (0.048)	0.024	0.53
Stride length left (m)	1.45 (0.147)	1.37 (0.115)	0.005	0.61
Stride length right (m)	1.45 (0.145)	1.38 (0.116)	0.009	0.53
% Stride length left (% height)	84.80 (24.486)	82.15 (7.045)	0.507	0.11
% Stride length right (% height)	84.72 (23.801)	82.61 (7.199)	0.589	0.09
Stance duration left (%)	58.62 (1.550)	59.36 (1.955)	0.045	−0.42
Stance duration right (%)	58.42 (1.830)	59.13 (2.084)	0.081	−0.36
Swing duration left (%)	41.38 (1.550)	40.64 (1.955)	0.045	0.42
Swing duration right (%)	41.58 (1.830)	40.87 (2.084)	0.081	0.36
First double support left (%)	8.62 (1.554)	9.06 (1.727)	0.198	−0.27
First double support right (%)	8.63 (1.586)	9.45 (2.128)	0.030	−0.44
Single support left (% cycle)	41.45 (1.703)	40.86 (2.013)	0.123	0.32
Single support right (% cycle)	41.36 (1.513)	40.88 (1.834)	0.163	0.29
Propulsion index left	8.14 (1.890)	9.38 (1.584)	0.001	−0.71
Propulsion index right	8.06 (1.935)	9.33 (1.495)	0.001	−0.73
Walk quality index left	96.61 (2.389)	96.86 (2.628)	0.638	−0.10
Walk quality index right	96.10 (2.832)	96.32 (2.593)	0.692	−0.08
Symmetry index of pelvic angles—tilt	70.52 (22.820)	67.48 (24.990)	0.539	0.13
Symmetry index of pelvic angles—obliquity	96.57 (8.609)	98.51 (0.612)	0.157	−0.32
Symmetry index of pelvic angles—rotation	97.37 (2.905)	96.93 (4.002)	0.535	0.13

**Table 3:** Paired *t*-test results comparing the left and right foot gait parameters.

Pair	Comparison	t-Value	*p*-Value	Effect Size (Cohen’s d)
Pair 1	Gait cycle duration left (s) vs. right (s)	1.727	0.087	0.04
Pair 2	Elaborated steps (number) left vs. right	2.367	0.020	0.24
Pair 3	Stride duration left (s) vs. right (s)	1.821	0.072	0.04
Pair 4	Stride length left (m) vs. right (m)	−1.372	0.173	−0.02
Pair 6	% Stride length left (% height) vs. % right (% height)	−1.053	0.295	−0.01
Pair 7	Stance duration left (%) vs. right (%)	1.388	0.169	0.11
Pair 8	Swing duration left (%) vs. right (%)	−1.388	0.169	−0.11
Pair 9	First double Support left (%) vs. right (%)	−1.115	0.268	−0.10
Pair 10	Single support left (%) vs. right (%)	0.280	0.780	0.03
Pair 11	Propulsion index left vs. right	0.660	0.511	0.04
Pair 12	Walk quality index left vs. right	1.938	0.056	0.20

## Data Availability

The original contributions presented in this study are included in the article. Further inquiries can be directed to the corresponding author.
